# The Role of Measurements Tools in the Association between Loneliness and Cognitive Decline

**DOI:** 10.1093/geroni/igaa057.3401

**Published:** 2020-12-16

**Authors:** Ji Hyang Cheon

**Affiliations:** University of Maryland, Baltimore, Maryland, United States

## Abstract

Previous findings on the association between loneliness and cognitive decline are mixed, with some studies indicating a significant association and others finding no statistical relationship. Since studies have used various inequivalent measurement tools, this might explain inconsistent findings on the relationship between loneliness and cognitive decline. This systematic review aims to 1) summarize the relationship between loneliness and cognitive decline and 2) examine whether the association varies depending on the measurement tools. This review’s inclusion criteria were studies with key terms loneliness as a predictor and global cognitive function as an outcome, peer-reviewed articles, written in English, involving community-dwelling older adults of ages 65 and older, and published since August 2013. Six out of ten studies showed that the relationship between loneliness and cognitive decline was statistically significant. Three reciprocal studies found that cognitive decline was a predictor of loneliness. Those studies that had nonsignificant findings used a single question to measure loneliness. Studies using the 23 item MMSE also reported non-significant association, whereas those using the 30 item MMSE had significant results. The findings indicate a bidirectional relationship derived from the studies with inconsistent results and the reciprocal studies and the potential role of measurement tools on the association’s change. This poster will conclude with the research implication that a systematic review of reciprocal studies is needed to clarify the bidirectional relationship of variables. Comparison studies of various measurement tools are needed to confirm the role of measurement tools on the result.

